# Age‐related deficits in neuronal physiology and cognitive function are recapitulated in young mice overexpressing the L‐type calcium channel, Ca_V_1.3

**DOI:** 10.1111/acel.13781

**Published:** 2023-01-26

**Authors:** Shannon J. Moore, Victor A. Cazares, Stephanie J. Temme, Geoffrey G. Murphy

**Affiliations:** ^1^ Michigan Neuroscience Institute Ann Arbor Michigan USA; ^2^ Molecular & Integrative Physiology University of Michigan Ann Arbor Michigan USA; ^3^ Department of Psychology Williams College Williamstown Massachusetts USA

**Keywords:** afterhyperpolarization, fear generalization, learning and memory, mouse, novel object recognition

## Abstract

The calcium dysregulation hypothesis of brain aging posits that an age‐related increase in neuronal calcium concentration is responsible for alterations in a variety of cellular processes that ultimately result in learning and memory deficits in aged individuals. We previously generated a novel transgenic mouse line, in which expression of the L‐type voltage‐gated calcium, Ca_V_1.3, is increased by ~50% over wild‐type littermates. Here, we show that, in young mice, this increase is sufficient to drive changes in neuronal physiology and cognitive function similar to those observed in aged animals. Specifically, there is an increase in the magnitude of the postburst afterhyperpolarization, a deficit in spatial learning and memory (assessed by the Morris water maze), a deficit in recognition memory (assessed in novel object recognition), and an overgeneralization of fear to novel contexts (assessed by contextual fear conditioning). While overexpression of Ca_V_1.3 recapitulated these key aspects of brain aging, it did not produce alterations in action potential firing rates, basal synaptic communication, or spine number/density. Taken together, these results suggest that increased expression of Ca_V_1.3 in the aged brain is a crucial factor that acts in concert with age‐related changes in other processes to produce the full complement of structural, functional, and behavioral outcomes that are characteristic of aged animals.

## Graphical abstract

Here we revisit the hypothesis that dysregulation of calcium homeostasis in neurons underlies age‐related changes in neuronal function and cognition. To test this hypothesis, we examined the post burst afterhyperpolarization and spatial memory in young mice genetically engineered to overexpress the L‐VGCC CaV1.3 in glutamatergic neurons in the hippocampus and forebrain.

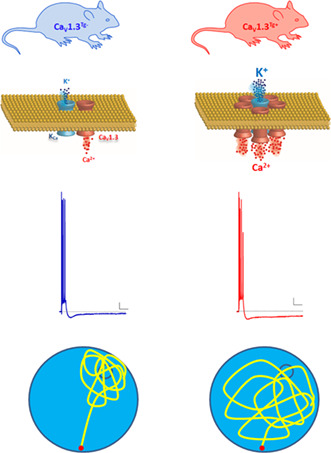

**Abbreviations**
aCSF: Artificial cerebrosprinal fluid
AOBS: Acousto‐optical beam splitter
AHP: Afterhyperpolarization
CFC: Contextual fear conditioning
fEPSPs: field Excitatory postsynaptic potentials
IR‐DIC: Infrared differential interference contrast
LTD: Long‐term depression
LTP: Long‐term potentiation
L‐VGCCs: L‐type voltage‐gated calcium channels
mAHP: Medium afterhyperpolarization
MWM: Morris water maze
NOR: Novel object recognition
NA: Numerical aperture
pAHP: Peak afterhyperpolarization
ROIs: Regions of interest
RM: Repeated measures
sAHP: Slow afterhyperpolarization
SR: Stratum radiatum
TQ: Target quadrant

## 1 INTRODUCTION

Aging is associated with impairments in cognitive function, particularly the ability to learn and remember new information. However, the neurobiological mechanisms that underlie this age‐related cognitive decline remain relatively poorly understood. Over four decades ago, Zaven Khachaturian advanced the idea that an age‐related change in the regulation of intracellular neuronal calcium ([Ca^2+^]_i_) leads to a variety of alterations in cellular physiology, ultimately resulting in a decline in cognitive function (Khachaturian, 1984, 1989). Experimental support for this “calcium dysregulation hypothesis” comes mostly from experiments in model organisms but it is also supported by a limited number of studies in humans (Birks & López‐Arrieta, 2002; Coon et al., 1999; Navakkode et al., 2018). Evidence from rodent studies was initially provided by a series of electrophysiological studies in which recordings from hippocampal CA1 neurons in aged animals (compared to those from young animals) exhibited an increased duration of calcium spikes, larger depolarization‐induced inward Ca^2+^ currents, and increased [Ca^2+^]_i_ (measured using a calcium‐sensitive fluorophore) in response to synaptically evoked action potential firing (Campbell et al., 1996; Hemond & Jaffe, 2005; Thibault & Landfield, 1996; Thibault et al., 2001).

In addition to these direct measurements of neuronal [Ca^2+^]_i_, age‐related alterations in many calcium‐dependent processes have also been observed. One of the most well‐documented changes is an age‐dependent reduction in intrinsic neuronal excitability, evidenced by an *increase* in the magnitude of the calcium‐sensitive component(s) of the postburst afterhyperpolarization (AHP). The AHP is a hyperpolarization of the membrane potential that occurs after a bout of high frequency action potential firing and lasts for several seconds before gradually returning to baseline. Experimentally and analytically, the AHP is often separated into different phases depending on kinetics and pharmacological sensitivity, which reflect characteristics of distinct underlying K^+^ currents (Gu et al., 2008; Mateos‐Aparicio et al., 2014; Sah & Faber, 2002; Sah, 1996). The fast component of the AHP (fAHP) lasts less than 10 ms and significantly contributes to the repolarization of the action potential; it is primarily mediated by voltage‐gated K^+^ channels (Sah, 1996) and does not typically exhibit age‐related changes in magnitude (Matthews et al., 2009). Conversely, the more prolonged phase of the AHP, which has an onset of several tens of milliseconds and lasts for several seconds, exhibits a pronounced Ca^2+^ dependence and has repeatedly been shown to be significantly larger in aged, compared to young, animals (including rabbits, rats, and mice) (Landfield & Pitler, 1984; Matthews et al., 2009; Moyer Jr. et al., 1992; Murphy et al., 2006; Power et al., 2002). Initially, this prolonged phase was thought to be mediated by a single Ca^2+^‐activated K^+^ conductance, but pharmacological studies have revealed two distinct underlying currents that can be used to further subdivide the AHP. A current blocked by the bee venom, apamin, mediates the intermediate (or medium) part of the AHP (mAHP); this current lasts tens to hundreds of milliseconds and is largely driven by the small conductance, Ca^2+^‐activated K^+^ (SK) channels. An even longer‐lasting part of the AHP (the slow AHP [sAHP]) is mediated by an apamin‐insensitive current, which has a slower rise time and lasts for several seconds. The exact identity of the channel (or channels) underlying this current remains somewhat controversial (Sahu & Turner, 2021), but it has a clear calcium dependence as pharmacological or genetic interventions that alter calcium levels strongly modulate its activity and, consequently, the size of the sAHP (Gamelli et al., 2011; McKinney et al., 2009). Thus, an age‐related increase in [Ca^2+^]_i_ is a likely driver of the age‐related increase in the sAHP via amplification of this underlying Ca^2+^‐activated K^+^ current.

Moreover, several studies have also demonstrated a link between these age‐related changes in calcium and calcium‐dependent processes and cognitive deficits in aged animals. For example, electrophysiological recordings from individual CA1 neurons in aged animals showed that the increase in Ca^2+^ current was due to an increase in channel number (as opposed to a change in channel function) and, importantly, that this increase in channel density correlated with the degree of impairment in a hippocampus‐dependent learning and memory task (the Morris water maze [MWM]) (Thibault & Landfield, 1996). Similarly, aged animals that have larger sAHPs also exhibit impairments in hippocampus‐dependent learning and memory tasks, like the MWM and trace eyeblink conditioning (Matthews & Disterhoft, 2009; Murphy et al., 2004, 2006; Tombaugh et al., 2005), and pharmacological treatments that reduce the sAHP facilitate learning and memory in these paradigms (Deyo et al., 1989; Kronforst‐Collins et al., 1997; Power et al., 2001; Weiss et al., 2000). Taken together, these data provide empirical support for the “calcium dysregulation hypothesis of brain aging” and suggest that an age‐related change in the regulation of intracellular calcium may initiate a cascade that modulates neuronal excitability and ultimately leads to cognitive decline in aged animals.

Many mechanisms are involved in regulating neuronal [Ca^2+^]_i_, including release from intracellular stores, sequestration by buffering proteins, and entry and extrusion through ion channels and pumps, respectively. Alterations to any of these processes are likely to disrupt calcium homeostasis and thus represent potential mediators of age‐related changes in neuronal [Ca^2+^]_i_ (Foster & Kumar, 2002; Murchison & Griffith, 2007; Thibault et al., 1998, 2007). One area of intense interest and investigation has focused on the role of L‐type voltage‐gated calcium channels (L‐VGCCs) (Moore & Murphy, 2020), which were originally differentiated from other channels because of their relatively *large* and *long‐lasting* (hence “L”‐type) calcium current (Tsien et al., 1991). With the advent of molecular cloning techniques, more detailed and precise characterization based on homology between primary pore‐forming (alpha) subunit(s) revealed that L‐VGCCs comprise 4 distinct subtypes (named Ca_V_1.1–Ca_V_1.4). In particular, Ca_V_1.2 and Ca_V_1.3 (also known as α_1C_ and α_1D_, respectively) are the predominant L‐VGCCs expressed in the nervous system, and while they differ partially in their biophysical properties (Xu & Lipscombe, 2001), they are difficult to functionally isolate in vivo (Lipscombe et al., 2004). Instead, investigators have used molecular biology techniques to interrogate the changes in expression of these isoforms that occur with age. In situ hybridization, immunohistochemistry, and Western blotting have shown that both Ca_V_1.3 mRNA and protein levels are increased in the hippocampus (particularly the CA1 region) of aged relative to young animals (Herman et al., 1998; Veng & Browning, 2002; Veng et al., 2003), while Ca_V_1.2 remains largely unaffected (although see (Herman et al., 1998)). Further, in individual CA1 neurons from aged animals, expression of Ca_V_1.3 mRNA directly correlates with the size of the L‐type calcium current recorded in those same neurons (Chen et al., 2000). Finally, the magnitude of the increase in Ca_V_1.3 protein directly correlates with the number of working memory errors in a hippocampus‐dependent task, and both this increased expression and impaired performance can be ameliorated by chronic treatment with nimodipine, a L‐VGCC blocker (Veng et al., 2003).

The wealth of data collected to date suggests that an age‐related increase in Ca_V_1.3 represents a common mechanism underlying dysregulation of neuronal [Ca^2+^]_i_, increased magnitude of the sAHP, and deficits in learning and memory that occur with age. However, these observations are correlative in nature; in fact, there are myriad differences in neuronal structure, function, and signaling that exist in parallel in aged animals, making it difficult to accurately elucidate specific causal relationships. Therefore, to directly assess the relative contribution of increased Ca_V_1.3 to altered functional outcomes, we generated a transgenic mouse line that has approximately 50% more Ca_V_1.3 protein in excitatory neurons in the forebrain (including the hippocampus) (Krueger et al., 2017). We have previously shown that these mice are generally normal, with no apparent deficits in sensory, motor, or anxiety‐like phenotypes (Krueger et al., 2017). Here we demonstrate that overexpression of Ca_V_1.3 *in young animals* (independent of other age‐related changes) recapitulates specific changes observed in aged animals; namely, an increase in the magnitude of the sAHP and impaired performance in hippocampus‐dependent learning and memory tasks (reference memory in the Morris water maze, novel object recognition, and contextual discrimination in fear conditioning). Thus, our results suggest that an increase in Ca_V_1.3 is sufficient to drive age‐related changes in both neuronal and cognitive function and, therefore, may represent a novel therapeutic target for the amelioration of age‐related cognitive decline.

## 2 MATERIALS AND METHODS

### 2.1 Mice

All procedures were approved by and performed in accordance with the University Committee on the Use and Care of Animals at the University of Michigan. We have previously described the generation of the novel mouse line (Ca_V_1.3^Tg+^) that expresses approximately 50% more Ca_V_1.3 protein in excitatory neurons in the forebrain and hippocampus, and showed that they exhibit normal sensory, motor, and anxiety‐related phenotypes (Krueger et al., 2017). For these studies, all mice were bred in‐house and maintained on a 14:10 h light–dark cycle with ad libitum access to food and water. Experimental groups consisted of young adult (3–9 months) wild‐type (Ca_V_1.3^Tg−^) and Ca_V_1.3^Tg+^ transgenic littermates on a C57BL/6Tac genetic background; investigators remained blind to genotype throughout all experiments. Separate cohorts of mice were used for the electrophysiological experiments and each of the different behavioral tests. Approximately equal numbers of males and females were included in each group (numbers are indicated in figure legends for specific experiments); because no statistically significant differences were found between sexes, data were combined and are presented here in aggregate.

### 2.2 Electrophysiology

#### 2.2.1 Slice preparation

Brain slices were prepared as previously described (Moore et al., 2011). Briefly, mice were first deeply anesthetized with isoflurane and then rapidly decapitated. The brain was removed and affixed to a stage submerged in ice‐cold, oxygenated (95% O_2_/5% CO_2_) sucrose cutting solution: (in mM): 206 sucrose, 2.8 KCl, 26 NaHCO_3_, 1.25 NaH_2_PO_4_, 1 CaCl_2_, 3 MgCl_2_, 10 D‐glucose, and 0.4 ascorbic acid. Coronal slices (250 μm; Leica VT1200S) were cut, bisected, and then transferred to a holding chamber containing room temperature artificial cerebral spinal fluid (aCSF; in mM): 125 NaCl, 2.5 KCl, 25 NaHCO_3_, 1.25 NaH_2_PO_4_, 2 CaCl_2_, 1 MgCl_2_, 25 D‐Glucose, and 0.4 ascorbic acid, for a minimum of 1 h prior to recording. Slices were individually transferred to a submersion style recording chamber and perfused (~1.5 ml/min) with oxygenated, heated (31–32°C) aCSF.

#### 2.2.2 Whole‐cell current‐clamp recordings

Pyramidal neurons in the CA1 region of the hippocampus were visualized using standard infrared differential interference contrast (IR‐DIC) optics and a Dage‐MTI NC‐70 camera. Glass pipettes fabricated from borosilicate filament glass (1.5 mm × 0.86 mm) with an open tip resistance of 4–8 MΩ contained a potassium methylsulfate‐based internal solution (in mM): 120 KMeSO_4_, 20 KCl, 2 MgCl_2_, 10 HEPES, 4 Na_2_‐ATP, 0.3 Tris‐GTP, 7 Tris‐phosphocreatine, and 0.2 EGTA, adjusted to a pH of 7.3 (with KOH) with an osmolarity of ~290–310 mOsm. Recordings were acquired at 50 kHz using a BVC 700A amplifier (Dagan Corporation), filtered at 5 kHz and digitized using an Axon 1322A Digidata (Molecular Devices) and a Dell desktop computer running pClamp 10 software (Molecular Devices). Neurons were accepted for recording if the resting membrane potential (Vm_rest_) was less than −55 mV at break‐in and action potentials displayed spike frequency accommodation (indicative of recording from pyramidal neurons, as opposed to interneurons). To ensure quality, series resistance (R_s_) and input resistance (R_in_) were monitored throughout the recording; cells were excluded from analysis if either measure changed more than 20%. There were no differences between groups in any of these properties (see Table 1). Recordings were not adjusted for the calculated liquid junction potential of +10.6 mV.

**TABLE 1:** Summary of Electrophysiological Parameters. In whole‐cell, current‐clamp experiments, pipette and passive electrophysiological properties were monitored to ensure high‐quality recordings. These included resting membrane potential at break‐in with no holding current (Vm_rest)_, current required to “clamp” the cell at −70 mV (I_hold_), input resistance (R_in_; measured throughout recording with a −50 pA, 250 ms hyperpolarizing current step), and pipette series resistance (R_s_; reported from amplifier and monitored throughout recording). There were no significant differences between groups on any measure (*p* > 0.05, unpaired *t*‐test between genotypes for each measure)

	Cells (#)	Mice (#)	Vm_rest_ (mV)	I_hold_ for ‐70 mV (pA)	R_in_ (MΩ)	R_s_ (MΩ)
Ca_V_1.3^Tg−^	9	3	−63.3 ± 1.6	−23.1 ± 6.1	163.2 ± 14.3	31.9 ± 2.4
Ca_V_1.3^Tg+^	13	4	−67.0 ± 1.8	−13.8 ± 12.1	192.6 ± 17.3	37.6 ± 4.1

The AHP was evoked from a holding potential of −60 mV with two protocols: (1) a 50 ms step current injection, at an amplitude sufficient to elicit five action potentials; or (2) a 1 s, 50 Hz train of step current injections (5 ms each), at an amplitude sufficient to elicit an action potential on each pulse in the train. Three successive sweeps were collected and averaged to determine the magnitude of the AHP. The voltage difference relative to the pre‐current injection holding potential was measured at three points to reflect different phases of the AHP (Sah, 1996): the peak AHP (pAHP) was the most hyperpolarized membrane potential reached after the current injection ended; the medium AHP (mAHP) was represented by the voltage deflection at 200 ms after the current injection ended; and the slow AHP (sAHP) was represented by the voltage deflection at 1000 ms after the current injection ended. In a subset of these recordings, a current/frequency (I/F) curve was also generated by counting the number of action potentials that were evoked by a 500 ms‐long step current injection from 0 to 500 pA (in 50 pA steps).

#### 2.2.3 Field potential recordings

Basal synaptic transmission was evaluated as previously described (Atkin et al., 2014; Murphy et al., 2006). Using a differential amplifier (DP‐301, Warner Instruments), extracellular recordings of field excitatory postsynaptic potentials (fEPSPs) were made from aCSF‐filled glass pipettes (tip resistances of ~1 MΩ) placed in the stratum radiatum (SR) of CA1 and fEPSPs were evoked by stimulating the Schaffer collateral afferent fibers with bipolar platinum electrodes (square pulse, 100 μs). Input/output curves were generated by delivering test stimuli once every 10 s with increasing intensity (from 0 to 0.5 mA).

#### 2.2.4 Primary hippocampal cell culture

Hippocampal cell cultures were prepared as previously described (Cazares et al., 2016). In brief, hippocampi from mice of either sex at postnatal day 1–2 were dissected and temporarily held in a HEPES‐buffered solution (HBS, GIBCO 14170–112, 10 mM HEPES added). Hippocampal tissue was partially digested via incubation in HBS containing 2 mg/ml papain (Sigma P3125) and 0.32 mg/ml L‐cystine (Sigma C7532) for 10 min at 37°C. A single cell suspension was then generated by pipette trituration, then pelleted via centrifugation (500 × G, 3 min), washed, resuspended, and plated on poly‐D‐lysine coated, 22 mm‐diameter, #1.5 thickness coverglass (Neuvitro, GG‐22). Cells were maintained in a 37°C, 95% O_2_/5% CO_2_ humidified incubator, and half the media was exchanged every 3–4 days with fresh NBActiv4 media (BrainBits).

#### 2.2.5 Immunocytochemistry

Immunocytochemistry was performed as described (Glynn & McAllister, 2006). Briefly, after 18–21 days in vitro, cell cultures were fixed (4% paraformaldehyde containing 4% sucrose), permeabilized (0.25% Triton‐X), blocked for nonspecific binding (10% bovine serum albumin), stained against MAP‐2 (SYSY 188003) and PSD‐95 (SYSY 124003), and visualized using species‐specific, spectrally distinct secondary antibodies (Alexa, Molecular Probes).

#### 2.2.6 Fluorescence imaging & image analysis

High‐resolution fluorescent images were acquired using an inverted laser‐scanning confocal microscope (Olympus Fluoview 1000), equipped with an acousto‐optical beam splitter (AOBS) and a tunable white‐light laser; accordingly, optical filter‐sets tuned for Alexa Fluor 488 (Ex: 488, Em: 498–584) and Alexa Fluor (Ex: 594, Em: 604–750) were used. Images were acquired with a 20x multi‐immersion objective or a 63× oil objective [Plan‐Apo, 1.4 numerical aperture (NA)] at a scanning rate of 200 Hz with 4x line averaging at 2× or 4× optical zoom. For each coverslip, three separate 2 × 2 tile series were captured. Each single frame in a tile series was made up of a 1024 × 1024 pixels (7.25 pixels per micron), per channel. All images were acquired with equal laser power and gain, set to achieve the full dynamic 16‐bit depth. Putative synaptic puncta were measured using FIJI (Schindelin et al., 2012), an open‐source distribution of ImageJ (https://imagej.nih.gov/ij/). First, single neuronal filaments (putative dendrites) were straightened using the “Straighten” plugin (https://imagej.net/plugins/straighten). Then, these single filament images were then auto‐thresholded based on fluorescence intensity, converted into a binary signal, and used to create regions of interest (ROIs) representing putative dendritic regions. Finally, the number of PSD95 puncta within these MAP2‐based ROIs was counted using the ImageJ “Analyze particles” plugin. Puncta per length measurements were averaged from 3–5 coverslips from each of three distinct cell culture preparations for each group (total coverslips: Ca_V_1.3^Tg−^, *n* = 10; Ca_V_1.3^Tg+^, *n* = 11).

### 2.3 Behavior

#### 2.3.1 Morris water maze (MWM)

Since its original description over 40 years ago (Morris, 1981), the Morris water maze has been used to study hippocampal‐dependent learning and memory with more than 11,000 publications cited in PubMed (Othman et al., 2022). With the advent of virtual reality technology, the MWM has been used extensively in humans (Thornberry et al., 2021) and has proved useful in studies where subjects exhibit sensory deficits (Dobbels et al., 2020). The MWM has also been used to evaluate cognitive deficits associated with AD (e.g., (Laczó et al., 2010; Possin et al., 2016)).The MWM experiments were conducted similarly to those previously described (McKinney & Murphy, 2006; White et al., 2008). The water maze consisted of a round white acrylic pool 1.2 m in diameter, which was filled with water heated to 27–28°C and made opaque using nontoxic white tempera paint. A round (hidden) platform made of clear acrylic measuring 10 cm in diameter was submerged just below the surface of the water in the northeast quadrant. Mice were tracked in two‐dimensional space in the water maze using a digital camera mounted above the pool. Digital tracking and off‐line analysis were performed with Actimetrics Water Maze 4 software (Wilmette, IL).

Mice were trained to find the platform in two sets of two back‐to‐back training trials with an interset interval of ~3–4 h (total of four trials per day) for 5 days. Each training trial began by placing an individual mouse on the platform for 15 s (to provide an opportunity to form a cognitive map of the room), after which they were put in the water facing the pool wall at pseudo‐random start locations. The time it took for each mouse to find the platform was recorded; if they did not reach the platform within 60 s, they were gently guided to the platform. Mice were allowed to remain on the platform for a period of 15 s at the end of each trial.

Spatial memory was assessed using probe trials (during which the platform was removed from the pool). Two probe trials were performed, with the first given prior to training at the beginning of Day 4 and the second on Day 6 (24 h after the last training trial on Day 5). The start position for each probe trial was in the southwest quadrant, opposite from the original platform location. Mice were allowed to swim for 60 s, after which they were removed from the pool, and the percent time spent searching each quadrant was recorded.

To ensure that mice did not exhibit any sensory, motor, or motivational deficits that might confound the interpretation of MWM, 24 h following the second probe trial, mice were given six visible platform trials (in three sets of two back‐to‐back trials, with an interset interval of ~1–2 h). The visible platform trials were performed as described for the (hidden platform) training trials, except that the platform was clearly marked with a flag and its location was moved for every set of two trials (to each of the quadrants except the original hidden platform quadrant).

#### 2.3.2 Novel object recognition (NOR)

The NOR task has been extensively used to investigate cognitive impairments in rodent models of neurological and psychiatric diseases (Grayson et al., 2015) and as a nonverbal cognitive measure in human subjects (e.g., Hampstead et al., 2010; Reynolds, 2015). The NOR experiments were carried out similarly to those previously published (Moore et al., 2013). The arena used for all trials was a 17‐gallon circular container made of white polyethylene, 42 cm high and 44.5 cm in diameter. The first day of each experiment consisted of 2–3 habituation trials (5 min each, 15–20 min apart) during which mice were exposed to the arena alone (no objects) in the training room. Twenty‐four hours later, the experimental trials began, which consisted of a familiarization phase and a test phase separated by a variable delay period. During the familiarization phase, mice were placed in the arena, which contained two copies of an object (constructed from LEGO® pieces), and allowed to freely explore (5 min per trial). After either a short (2 min) or extended (20 min) delay period, a test trial (5 min) was conducted; mice were returned to the arena, which contained one of the original objects (“familiar”) and a new, different object (“novel”). The object assignments (familiar or novel) and locations (left or right side of the arena) were counterbalanced within each experiment. Objects were placed ~10 cm from the arena wall and the arena and objects were cleaned between each phase and between trials with 70% ethanol. Importantly, all groups of mice explored objects during the familiarization and test phases for at least 50 s, and there were no differences between groups in total exploration time (data not shown).

#### 2.3.3 Fear generalization/context discrimination

We modified published protocols for fear conditioning (Cazares et al., 2019; Temme et al., 2014) to test the effect of exposure to different environments on fear generalization and context discrimination. In the first experiment, (*Training* vs *Novel Context*), mice were trained for 3 consecutive days (Day 1–3) in Context A, which consisted of metal grid floors, transparent square walls, white lights, and a 70% ethanol scent. Each training day consisted of one trial with a 180 s baseline period, followed by a single unsignaled foot shock (2 s, 0.75 mA) delivered through the grid floor, and a 60 s post‐shock period, after which mice were removed from the chamber and returned to their home cage. Twenty‐four hours after the last day of training (on Day 4), mice were re‐exposed to Context A for 3 min (with no foot shock). Twenty‐four hours later (on Day 5), mice were placed in a novel context (Context C, which had a solid white acrylic floor, curved white acrylic walls, red lights, and a 2% acetic acid scent) for 3 min (also with no foot shock). In the second experiment, (*Similar* vs *Novel Context*) mice were trained as described above, except that mice received three shocks per day (30 sec ISI) and on Day 4, the mice were placed in a context that was similar to the training context for 3 min (called Context B, in which only the flooring differed by covering the metal grid with a cushioned mat liner). On Day 5, the mice were placed in Context C for 3 min, as in the first experiment. Video recording of all trials and analysis of percent time freezing was performed by FreezeFrame 4 software (Actimetrics).

### 2.4 Statistical analysis

All analysis was performed using GraphPad Prism 8.0. All average data is presented as mean ± sem. Appropriate statistical tests were selected for each experiment (as indicated in figure legends for specific experiments), and include: unpaired two‐tailed Student's *t*‐tests (for comparisons between two groups); mixed model ANOVA with planned post‐hoc comparisons (for multigroup or multifactor comparisons) and/or repeated measures ([RM], for multiple time points); or one‐tailed Student's *t*‐tests (for comparison against “chance” [25%] performance in MWM probe trials). Statistical significance was considered as *p* < 0.05.

## 3 RESULTS

**FIGURE 1:**
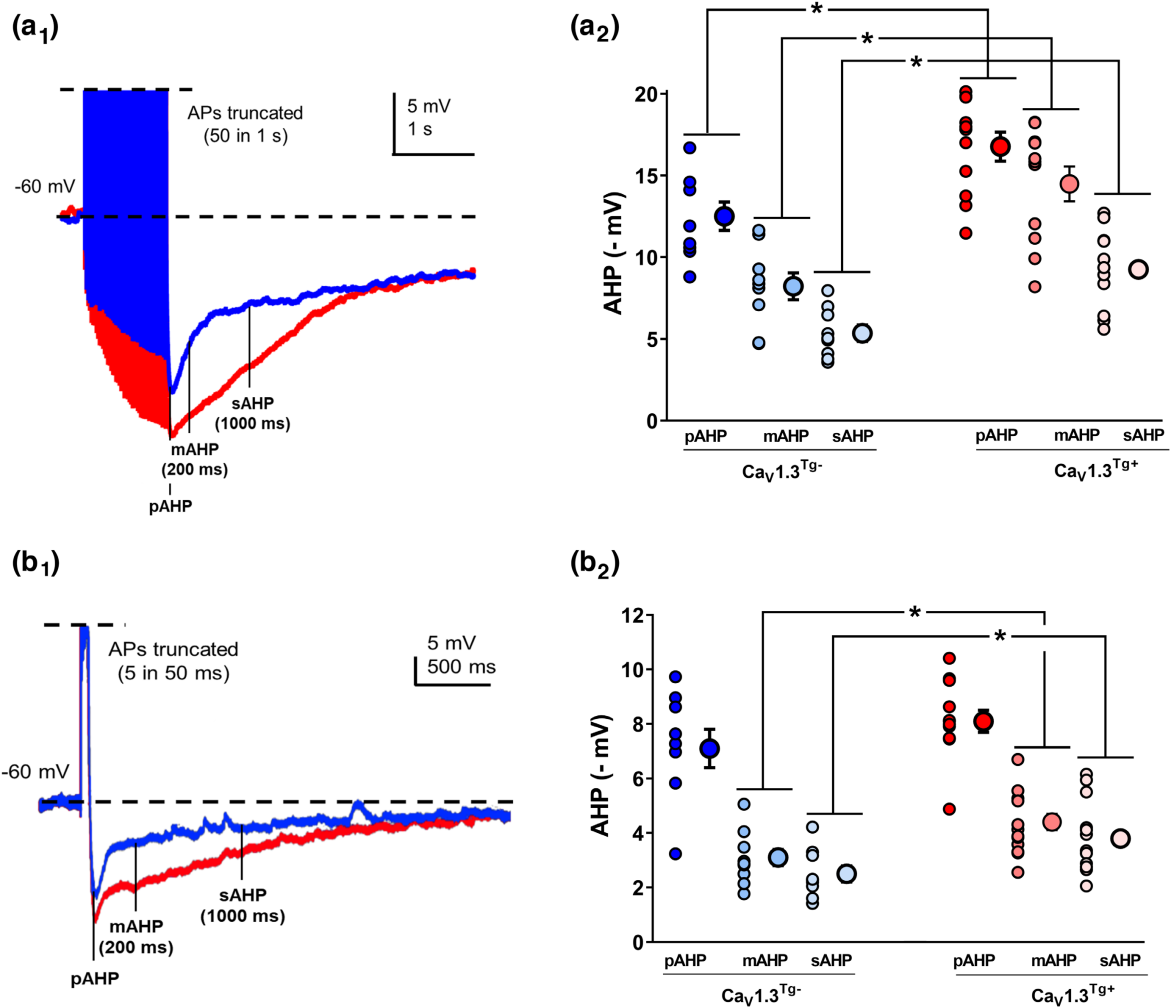
Overexpression of Ca_V_1.3 increases the amplitude of the postburst AHP. Whole‐cell current‐clamp recordings were made in pyramidal neurons from CA1 hippocampus in ex vivo slices prepared from young (3–6 months) Ca_V_1.3^Tg−^ and Ca_V_1.3^Tg+^ mice (Ca_V_1.3^Tg−^, *n* = 9 cells from three animals; Ca_V_1.3^Tg+^, *n* = 13 cells from four animals). (a_1_) Representative examples of the postburst AHP elicited by a 1 s train of action potentials at 50 Hz. The amplitude of the peak AHP (pAHP), medium AHP (mAHP) and slow AHP (sAHP) were quantified at time points indicated. (a_2_) This supraphysiological stimulation protocol induced a sizable AHP in both groups; however, all phases of the AHP (including pAHP, mAHP and sAHP) were significantly enhanced in the Ca_V_1.3^Tg+^ mice (*p* < 0.001 main effect of genotype, 1‐way ANOVA; * indicates *p* < 0.05, planned post‐hoc comparisons). (b_1_) Representative examples of the postburst AHP elicited by a 50 ms depolarizing step sufficient to elicit five action potentials (action potentials truncated for clarity). (b_2_) Using this more physiological stimulation protocol, only the mAHP and sAHP were significantly increased by overexpression of Ca_V_1.3 (*p* < 0.001 main effect of genotype, 1‐way ANOVA; * indicates *p* < 0.01, planned post‐hoc comparisons). In both (a_2_) and (b_2_), small circles represent measurements from individual neurons while large circles reflect averaged data (mean ± sem).

To determine to what extent Ca_V_1.3 overexpression impacted the postburst AHP, whole‐cell current‐clamp recordings were made from CA1 hippocampal neurons in ex vivo slices prepared from young Ca_V_1.3^Tg−^ and Ca_V_1.3^Tg+^ mice (Figure 1). We first used a strong protocol to evoke the AHP, in which a train of brief current injections elicited 50 action potentials in 1 s (Figure 1a_1_). This supraphysiological stimulation elicited a large AHP in both groups; however, all phases of the AHP (pAHP, mAHP & sAHP) were significantly larger in Ca_V_1.3^Tg+^ cells (Figure 1a_2_). We next turned to a more physiological protocol that utilized a single depolarizing step (50 ms) at an amplitude just sufficient to elicit 5 action potentials (Figure 1b_1_). In this case, only the mAHP and the sAHP were enhanced in the Ca_V_1.3^Tg+^ mice (Figure 1b_2_), which is consistent with previous work suggesting that calcium gated by L‐VGCCs preferentially modulates the later phases of the postburst AHP (Lima & Marrion, 2007; Power et al., 2002; Sahu et al., 2017).

**FIGURE 2:**
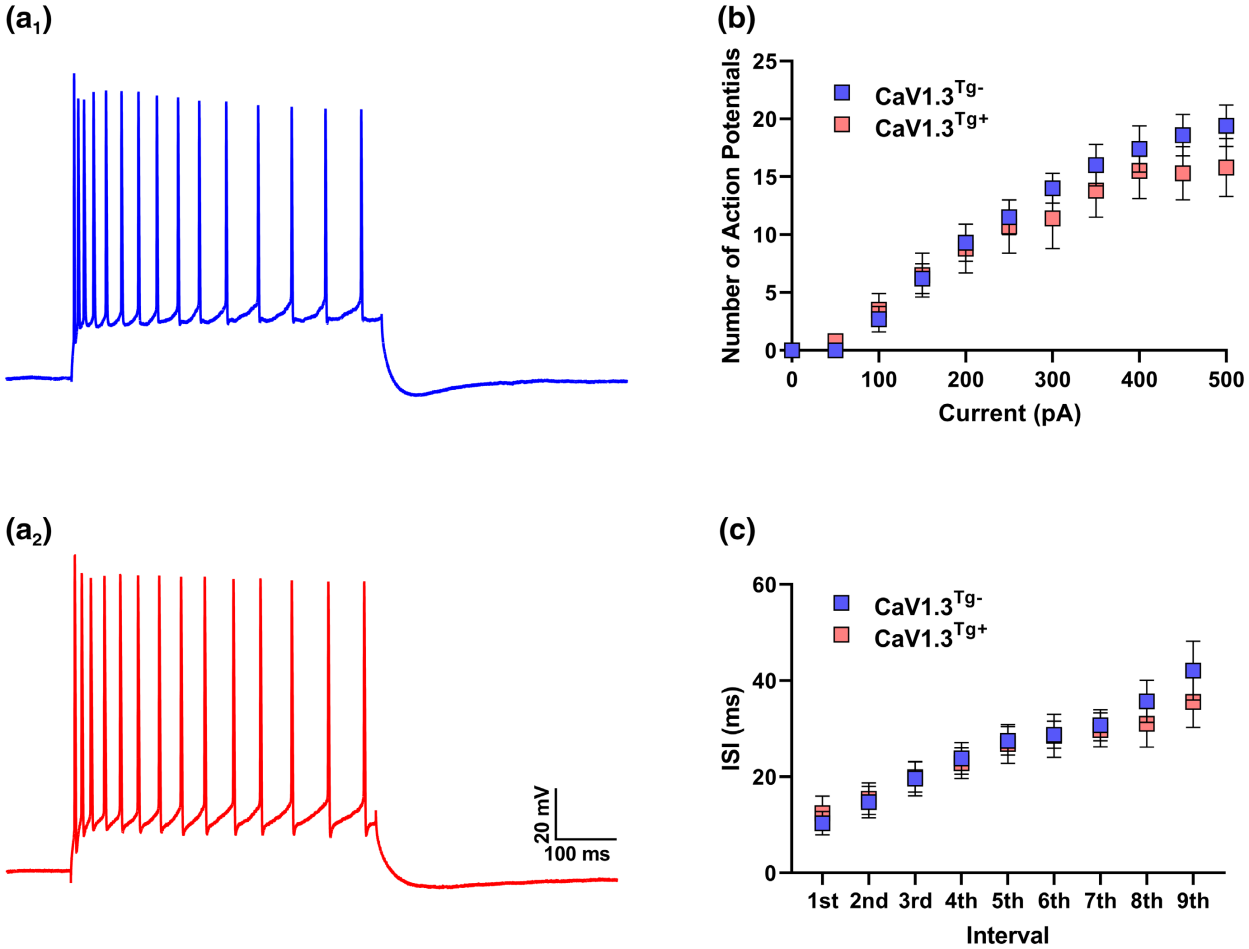
Overexpression of Ca_V_1.3 does not affect repetitive firing in CA1 pyramidal neurons. In a subset of neurons in which the postburst AHP was recorded (see Figure 1), repetitive action potential firing was also examined (Ca_V_1.3^Tg−^, *n* = 6; Ca_V_1.3^Tg+^, *n* = 6), using a series of 500 ms‐long step current injections with amplitudes from 0 to 500pA in 50pA increments. (a) Representative traces from Ca_V_1.3^Tg−^ (a_1_) and Ca_V_1.3^Tg+^ (a_2_) neurons show the voltage response to the 300‐pA step. (b) Neurons from both genotypes exhibited a significant increase in the number of action potentials elicited with increasing current injections, but there was no difference between groups (*p* < 0.001 main effect of current, *p* > 0.05 main effect of genotype, 2‐way RM ANOVA). (c) In addition, neurons from both genotypes also exhibited an increase in the interspike interval (ISI) over the duration of the current injection (spike frequency accommodation is distinguishing characteristic of CA1 pyramidal neurons), but again there were no significant difference between the groups (*p* < 0.001 main effect of current, *p* > 0.05 main effect of genotype, 2‐way RM ANOVA). Data in (b) and (c) are presented as mean ± sem.

Canonically, an *increase* in the size of the postburst AHP has been thought to affect functional outcomes by *decreasing* the number and/or frequency of action potential firing (Gant & Thibault, 2009; Moyer Jr. et al., 1992; Oh et al., 1999), but, intriguingly, some studies have shown that these properties can be dissociated (Gamelli et al., 2011; Joëls & de Kloet, 1989; Liebmann et al., 2008). Thus, to investigate the role of Ca_V_1.3 overexpression on repetitive firing, we used a family of step current injections (from 0 to 500pA, 500 ms) to probe the current/frequency (I/F) relationship (Figure 2). Neurons from both Ca_V_1.3^Tg+^ and Ca_V_1.3^Tg−^ mice exhibited spike frequency accommodation, characteristic of pyramidal neurons in the CA1 region of the hippocampus (Figure 2a), and both responded to increasing current injection amplitude with an increasing number/frequency of action potentials (Figure 2b). Interestingly, however, despite the significant difference in the size of the AHP in these groups, there was no difference in the I/F relationship. Similarly, there was no difference in accommodation as measured by the interspike interval of the first ten spikes (Figure 2c). This data is consistent with our previous work demonstrating that genetic ablation of Ca_V_1.3 significantly reduced the size of the AHP but had no effect on action potential firing (Gamelli et al., 2011). Taken together, these data suggest that these two aspects of neuronal excitability can be decoupled in CA1 hippocampus, and, further, that Ca_V_1.3 is preferentially involved in regulating the AHP but not repetitive firing.

**FIGURE 3:**
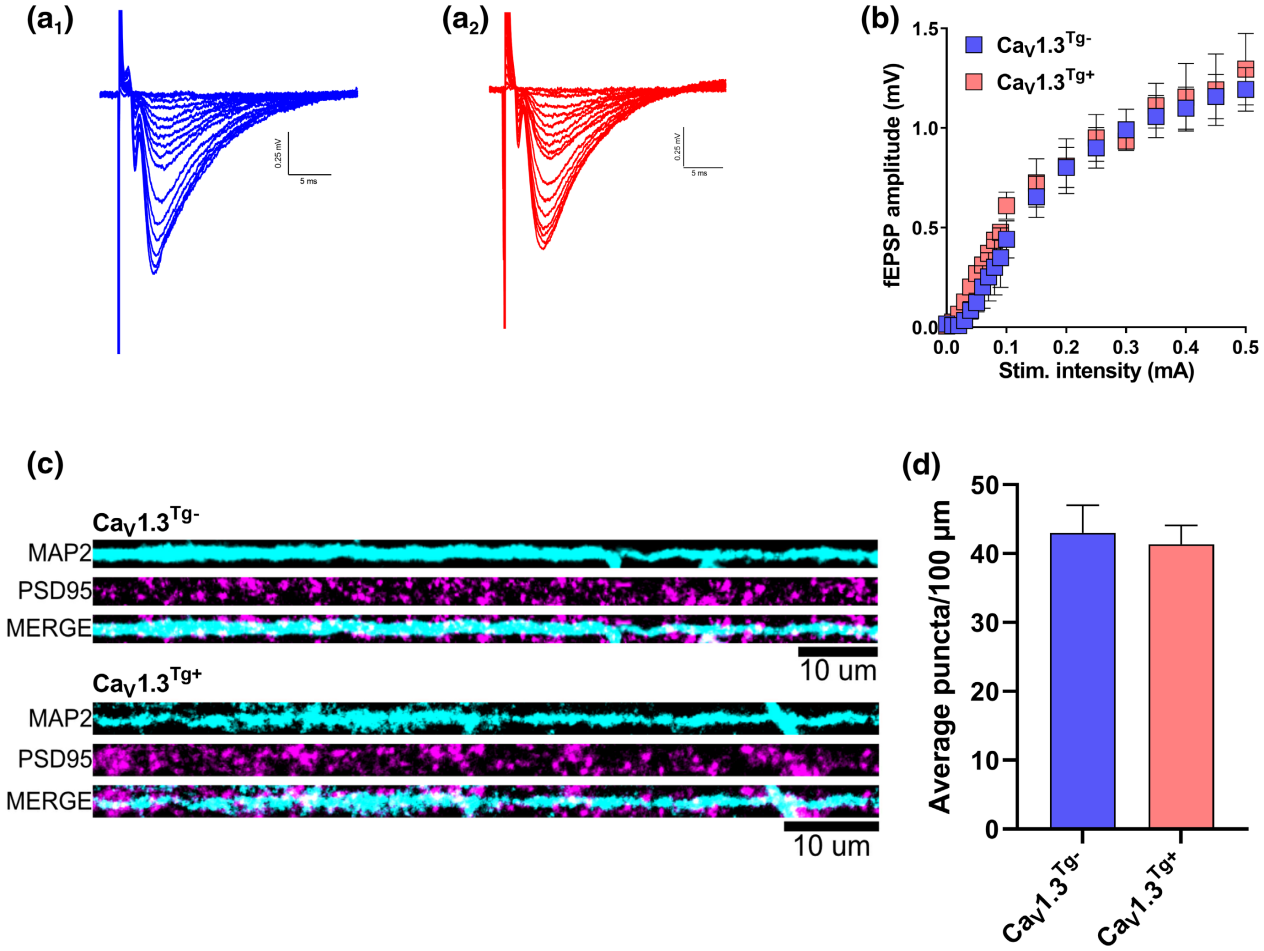
Excitatory synapses are not altered in Ca_V_1.3^Tg+^ mice. (a) Field excitatory postsynaptic potentials (fEPSPs) were recorded by placing extracellular stimulating and recording electrodes in stratum radiatum (SR) of CA1 in ex vivo hippocampal slices prepared from Ca_V_1.3^Tg−^ and Ca_V_1.3^Tg+^ mice (Ca_V_1.3^Tg−^, *n* = 5 slices from three animals; Ca_V_1.3^Tg+^, *n* = 3 slices from two animals). (a) Representative traces from Ca_V_1.3^Tg−^ (a_1_) and Ca_V_1.3^Tg+^ (a_2_) neurons. (b) Average amplitudes of the fEPSPs are plotted as a function of stimulation intensity to generate input/output (I/O) curves. Both groups exhibited typical I/O curves with fEPSP amplitudes increasing in proportion to stimulation strength; there was no significant difference between genotypes (*p* < 0.001 main effect of stimulation intensity, *p* > 0.05 main effect of genotype, 2‐way RM ANOVA). (c) Representative images from in vitro neuronal cultures, prepared from hippocampi isolated from Ca_V_1.3^Tg−^ and Ca_V_1.3^Tg+^ mice and stained for MAP2 and PSD95. (d) Confocal images were acquired to quantify the number of dendritic PSD95^+^ puncta (Ca_V_1.3^Tg−^, *n* = 10 coverslips from three preps; Ca_V_1.3^Tg+^, *n* = 11 coverslips from three preps). Consistent with our neurophysiological findings, Ca_V_1.3^Tg+^ mice exhibited no difference in the average number of puncta (*p* > 0.05, unpaired t‐test) suggesting that overexpression of Ca_V_1.3 does not alter synaptic connectivity within the CA1 region of the hippocampus. Data are presented as mean ± sem.

Previous studies have demonstrated multiple age‐related changes in synaptic architecture and function, including a decrease in the number of excitatory synapses (assessed by the number of PSD95‐positive puncta and/or dendritic spine density), decreased basal synaptic communication (reflected as a decrease in the amplitude of the excitatory postsynaptic response to the same pre‐synaptic stimulation), and a bias towards less synaptic potentiation/more synaptic depression (i.e., decreased long‐term potentiation [LTP] and increased long‐term depression [LTD]) (for reviews, see (Barnes, 1994; Foster, 1999; Kelly et al., 2006; Lynch, 1998; Rosenzweig & Barnes, 2003)). Because calcium and calcium signaling affects many of these processes, it is important to assess the effect of Ca_V_1.3 overexpression on excitatory synaptic function. Therefore, we prepared ex vivo hippocampal slices from Ca_V_1.3^Tg+^ and Ca_V_1.3^Tg−^ mice and recorded field excitatory postsynaptic potentials (fEPSPs) in CA1 stratum radiatum (SR) in response to Schaffer collateral stimulation (Figure 3). Synaptic responses (measured as fEPSP amplitude) to increasing stimulation strength (Figure 3a) were used to generate input/output (I/O) curves. As illustrated in the averaged I/O curves (Figure 3b), overexpression of Ca_V_1.3 does not alter basal (excitatory) synaptic transmission. Consistent with this finding, we also did not observe any significant difference in the number of excitatory synapses (as indicated by labeling PSD95‐positive puncta) in neurons cultured from hippocampi of Ca_V_1.3^Tg−^ or Ca_V_1.3^Tg+^ mice (Figure 3c,d).

**FIGURE 4:**
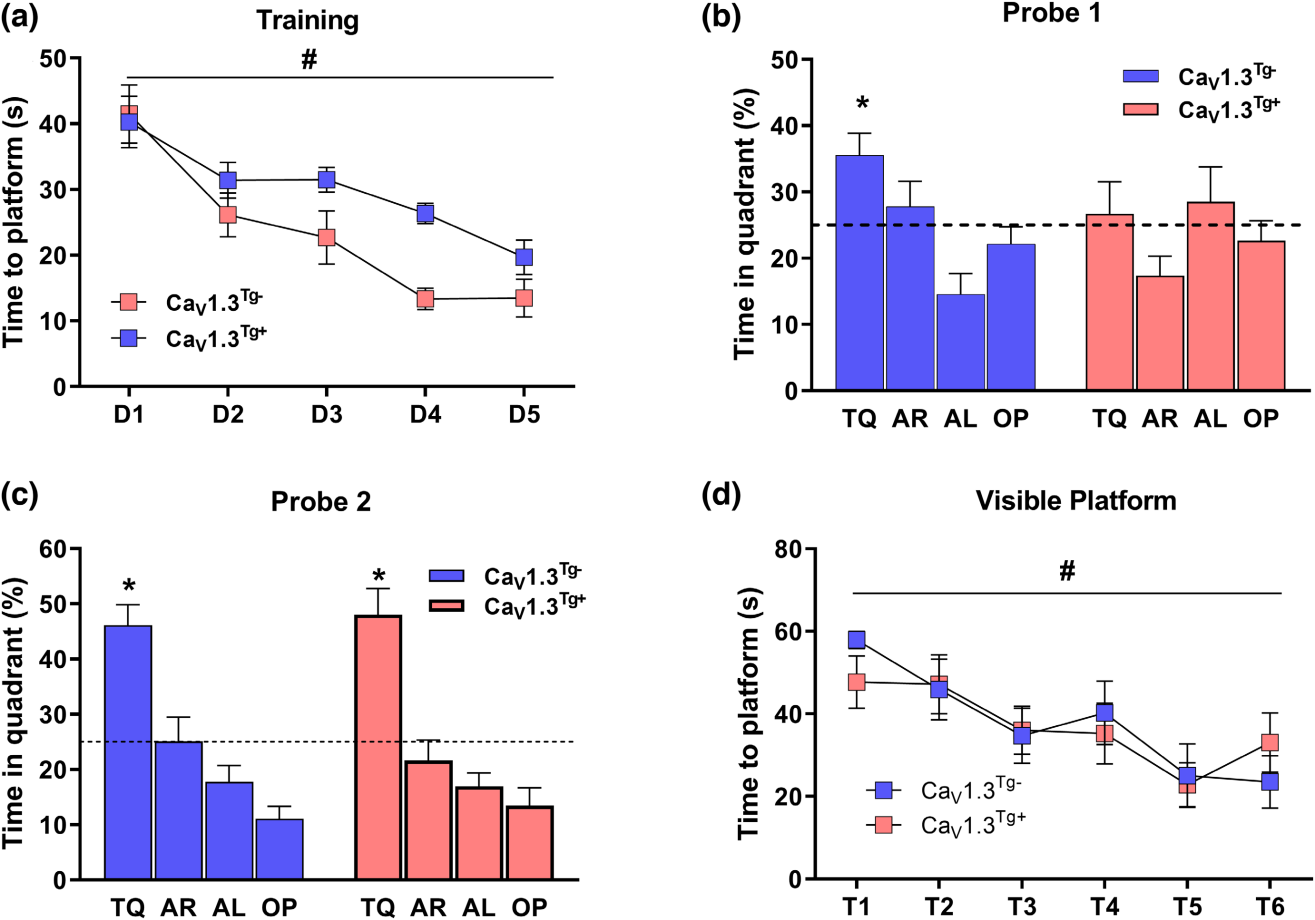
Overexpression of Ca_V_1.3 results in aging‐like spatial memory deficits in the Morris water maze (MWM). (a) Training consisted of four trials per day (D1‐D5) in which mice (Ca_V_1.3^Tg−^, *n* = 8; Ca_V_1.3^Tg+^, *n* = 9) were placed at random locations around the edge of the pool and allowed to swim for 60 s or until they found the platform, which was hidden just below the surface of the water. The latency to locate the hidden platform significantly decreased across training days with no statistically significant differences between groups (# indicates *p* < 0.001 main effect of training, *p* > 0.05 main effect of genotype, 2‐way RM ANOVA). (b,c) Two probe trials (60 s) were given to assess memory formation; in these trials, the platform was removed from the pool and the percent time mice spent swimming in the quadrant where the platform was previously located (target quadrant, TQ) was measured, compared to the adjacent right (AR), adjacent left (AL), and opposite (OP) quadrants. (b) In the first probe trial, conducted before training on Day 4, Ca_V_1.3^Tg‐^ mice exhibited a selective search strategy (*p* < 0.001 main effect quadrant, 1‐way ANOVA) and spent a significant proportion of time in the TQ (* indicates *p* < 0.05, 1‐sample *t*‐test against chance performance level [25%, dashed line in figure]), suggesting that they had already successfully formed a memory for the platform location. Conversely, Ca_V_1.3^Tg+^ mice exhibited a random search strategy (*p* > 0.05 main effect quadrant, 1‐way ANOVA) that was not different from the “chance” performance level (*p* > 0.05, 1‐sample *t*‐test against 25%) demonstrating that their memory formation was impaired. (c) In the second probe trial (conducted on Day 6, 24 h after the final training trial), Ca_V_1.3^Tg−^ mice continued to demonstrate memory for the platform location (*p* < 0.001 main effect quadrant, 1‐way ANOVA and * indicates *p* < 0.05, 1‐sample *t*‐test against 25%). Importantly, Ca_V_1.3^Tg+^ mice now also exhibited memory for the platform location, reflected by a selective search strategy (*p* < 0.001 main effect quadrant, 1‐way ANOVA) and a significant proportion of time spent in the TQ (* *p* < 0.05, 1‐sample t‐test against 25%). (d) To ensure that the overexpression of Ca_V_1.3 did not impact other functions (such as sensory, motor, or motivational aspects) that could confound interpretation of learning and memory performance, 24 h after the second probe trial (on Day 7), mice received six trials (T1–T6) on the hippocampus‐independent version of the MWM. These “visible platform” trials were performed exactly as described for the hidden platform training trials except that the platform was clearly marked with a visible flag. Both groups quickly swam to the marked platform (# indicates *p* < 0.001 main effect of trial number, *p* > 0.05 main effect of genotype, 2‐way RM ANOVA), suggesting that the learning and memory impairments observed in the Ca_V_1.3^Tg+^ mice were principally mediated by alterations in cognitive function. All data are presented as mean ± sem.

Age‐related changes in the sAHP have been linked to cognitive impairments in rabbits, rats and mice (for reviews, see (Disterhoft & Oh, 2007; Dunn & Kaczorowski, 2019; Foster, 2019; Moore & Murphy, 2020; Oh et al., 2010)).To determine to what extent the overexpression of Ca_V_1.3 (and the resulting enhanced sAHP) might impact cognitive function, Ca_V_1.3^Tg−^ and Ca_V_1.3^Tg+^ mice were evaluated in several learning and memory tasks, which are known to be sensitive to aging. We first assessed hippocampal‐dependent learning and memory using the Morris water maze (MWM) (Figure 4). Mice were trained to find a hidden escape platform over the course of 5 days (4 trials/day) and the amount of time required to reach the platform (latency) was used as a measure of spatial learning (Figure 4a). Both Ca_V_1.3^Tg−^ and Ca_V_1.3^Tg+^ mice exhibited a significant reduction in latency across days of training (indicating that both groups learned), but there was no significant difference between the groups. To assess spatial reference memory, probe trials (in which the platform was removed) were conducted before the start of training on Day 4 (Probe 1) and 24 h after the last training trial (Probe 2). During the first probe trial (Figure 4b), the Ca_V_1.3^Tg−^ mice preferentially searched for the platform in the quadrant of the pool where it was previously located (target quadrant, [TQ]), suggesting they had already formed a memory for the platform location. Conversely, the Ca_V_1.3^Tg+^ mice displayed a random search strategy in which their time spent in the TQ was not significantly above chance (Figure 4b). However, in the second probe trial, Ca_V_1.3^Tg+^ mice show significantly improved performance and also displayed a selective search strategy for the TQ, suggesting they were able to eventually form a memory for the platform location (Figure 4c). This finding is especially interesting because it is reminiscent of the performance of aged wild‐type mice in the MWM in which they take longer to learn (require more training trials) than do young mice, but with additional training, they form robust memories for the platform location (Murphy et al., 2004). Importantly, the deficits observed in the Ca_V_1.3^Tg+^ mice were not confounded by changes in other noncognitive processes (such a sensory, motor, or motivational aspects), as both Ca_V_1.3^Tg−^ and Ca_V_1.3^Tg+^ mice performed equally well on the hippocampal‐independent version of the MWM (the “visible platform”, in which the platform is clearly marked; Figure 4d).

**FIGURE 5:**
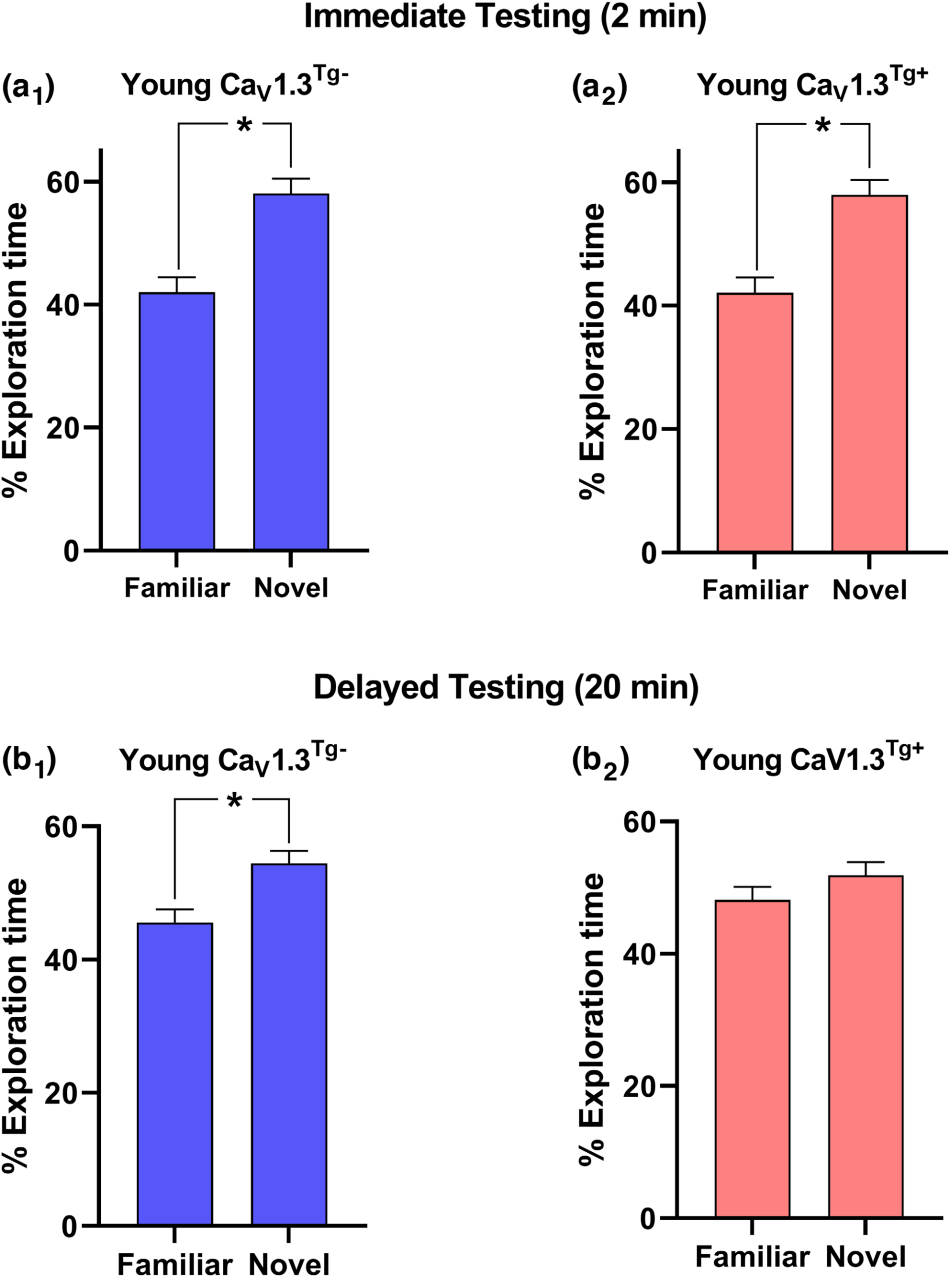
Overexpression of Ca_V_1.3 results in aging‐like memory deficits in novel object recognition (NOR). For NOR experiments, mice were first placed in an open arena and allowed to explore two objects that were identical in shape, size, and color for a total of 5 min (“familiarization phase”). Following a delay period (in which mice were placed in a holding cage), mice were returned to the arena for 5 min and allowed to explore two objects, one that was identical to the previously explored objects (the “familiar” object) and a new object to which the mice had not been previously exposed (the “novel” object). Short‐term memory was assessed by comparing the amount of time spent exploring the objects; significantly more time exploring the novel object was taken to be indicative of a memory for the previous exposure to the familiar object. (a) *Immediate Testing*: When the delay period was very short (2 min), both Ca_V_1.3^Tg−^ (a_1_; *n* = 15) and Ca_V_1.3^Tg+^ (a_2_; *n* = 17) mice spent significantly more time exploring the novel object (* indicates *p* < 0.01, paired t‐test), suggesting both remembered the previous exposure to the familiar object. (b) *Delayed Testing*: In a separate experiment, Ca_V_1.3^Tg−^ (b_1_; *n* = 13) and Ca_V_1.3^Tg+^ (b_2_; *n* = 12) mice were given a longer delay period (20 min). In this case, Ca_V_1.3^Tg−^ mice still exhibited a preference for the novel object (* indicates *p* < 0.05, paired *t*‐test), suggesting they had intact short‐term memory. However, Ca_V_1.3^Tg−^ mice spent similar amounts of time exploring both objects (*p* > 0.05, paired *t*‐test), demonstrating an impairment in their short‐term memory. All data are presented as mean ± sem.

Next, we examined short‐term memory using the novel object recognition (NOR) paradigm which in mice, requires an intact hippocampus (Cinalli Jr. et al., 2020; Cohen et al., 2013; Stackman et al., 2016). In this task, mice were first allowed to explore two identical objects during the familiarization phase. After a delay period (in which mice were removed from the arena and placed in a holding cage), mice were returned to the arena for the test phase in which they had the opportunity to explore the object from the familiarization phase (“familiar” object) or a completely new object (“novel” object) (Figure 5). Both Ca_V_1.3^Tg−^ and Ca_V_1.3^Tg+^ mice spent significantly more time exploring the novel object during the test phase when the delay was short (2 min; Figure 5a_1_,a_2_), suggesting that both groups formed a memory for the familiar object. When the delay was extended to 20 min, Ca_V_1.3^Tg−^ still spent significantly more time exploring the novel object, but Ca_V_1.3^Tg+^ mice spent equal time exploring both the familiar and novel object (Figure 5b_1_,b_2_), demonstrating a deficit in memory for the familiar object. These results parallel and synergize with NOR experiments in aged mice: wild‐type mice (Ca_V_1.3^+/+^)exhibit an age‐related impairment in short‐term memory (no preference for the novel object after a 20 min delay period), but if Ca_V_1.3 is genetically ablated (Ca_V_1.3^−/−^), short‐term memory is intact, with aged Ca_V_1.3^−/−^ mice spending more time exploring the novel object (Figure S1).

**FIGURE 6:**
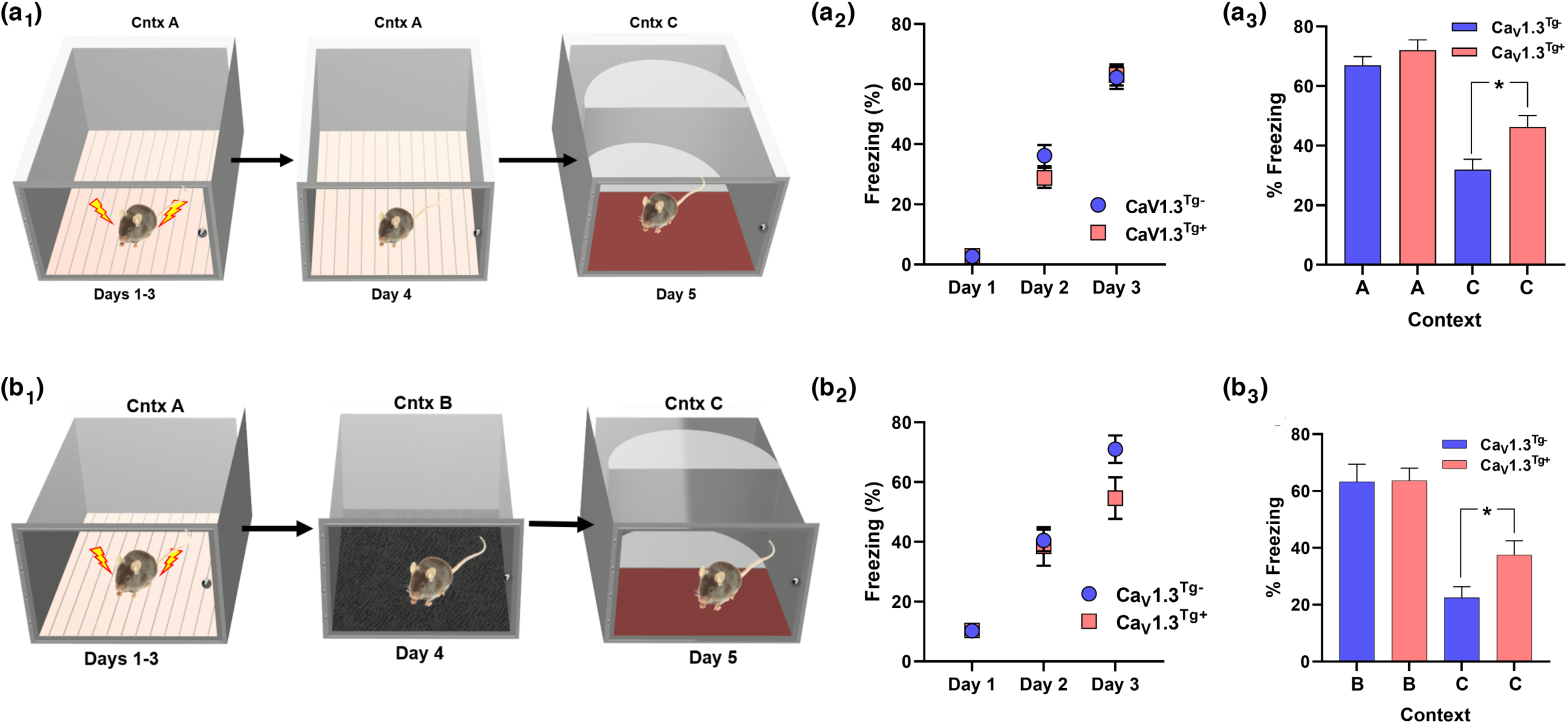
Overexpression of Ca_V_1.3 results in an aging‐like increase in fear generalization. Once per day for 3 days, mice were individually placed in the training context (Context A) for 180 s before receiving one unsignaled foot shock (2 s, 0.75 mA); after an additional 60 s, mice were removed from the fear conditioning chamber and returned to their home cage. Subsequently, context tests (24 h apart) in which freezing was measured for 180 s (in the absence of a foot shock) were carried out in the same training context (Context A), a context similar but not the same as the training context (Context B; the metal grid floor was changed to a cushioned mat floor), or a completely novel context (Context C). (a) *Training* versus *Novel Context*: (a_1_) Ca_V_1.3^Tg−^ (*n* = 30) and Ca_V_1.3^Tg+^ (*n* = 18) mice were trained as described above. (a_2_) Freezing levels, measured during the 180 s prior to the foot shock, increased across the 3 days of training in both groups, but there were no significant differences between the genotypes (*p* < 0.0001 main effect of training, *p* > 0.05 main effect of genotype, 2‐way RM ANOVA). (a_3_) Twenty‐four hours after the final training session (on Day 4), mice were returned to the training context (Context A); both Ca_V_1.3^Tg‐^ and Ca_V_1.3^Tg+^ mice exhibited similarly high levels of freezing (indicating that they formed an association between that context and an aversive outcome). After another 24 h (on Day 5), mice were tested in a completely novel context (Context C); Ca_V_1.3^Tg+^ mice froze significantly more compared to Ca_V_1.3^Tg−^ mice (*p* < 0.0001 main effect of context, 2‐way RM ANOVA; * indicates *p* < 0.05, planned post‐hoc comparisons), showing that they overgeneralize their fear memory and cannot distinguish between disparate contexts. (b) *Similar* vs *Novel Context*: (b_1_) In a separate experiment, Ca_V_1.3^Tg−^ (*n* = 11) and Ca_V_1.3^Tg+^ (*n* = 9) mice were trained as described above, except that they received three‐foot shocks (2 s, 0.75 mA; intershock interval 30 s) each day for 3 days. (b_2_) As before, freezing levels increased across the 3 days of training but there were no significant differences between the genotypes (*p* < 0.0001 main effect of training, *p* > 0.05 main effect of genotype, 2‐way RM ANOVA). (b_3_) Twenty‐four hours after the final training session (on Day 4), mice were placed in a context similar to the training context (Context B); again, both Ca_V_1.3^Tg−^ and Ca_V_1.3^Tg+^ mice exhibited similarly high levels of freezing. After another 24 h (on Day 5), mice were tested in a completely novel context (Context C); again, Ca_V_1.3^Tg+^ mice froze significantly more compared to Ca_V_1.3^Tg‐^ mice (*p* < 0.0001 main effect of context, 2‐way RM ANOVA; * indicates *p* < 0.05, planned post‐hoc comparisons), showing that the overgeneralization of fear memory is not due to the effect of re‐exposure to the training context and instead that Ca_V_1.3^Tg+^ mice have a pervasive impairment in context discrimination. Data are presented as mean ± sem.

While there are conflicting reports regarding the ability of aged rodents to exhibit associative learning in contextual fear conditioning (CFC) paradigms, with some showing performance equal to that of young animals (Aziz et al., 2019; Blank et al., 2003; Gould & Feiro, 2005; McAvoy et al., 2016; Sanders, 2011) but others finding age‐related deficits (Clausen et al., 2010; Peleg et al., 2010; Peters et al., 2014), an age‐related increase in fear generalization has been consistently observed (Kaushik et al., 2018; McAvoy et al., 2016; Shoji & Miyakawa, 2019). This age‐related increase in fear generalization likely contributes to deficits in context discrimination observed in aged rodents (Wu et al., 2015), which is akin to an age‐related deficit in pattern discrimination observed in humans (Yassa & Stark, 2011; Yassa et al., 2011). Therefore, we investigated context‐dependent fear generalization in Ca_V_1.3^Tg−^ and Ca_V_1.3^Tg+^ mice (Figure 6). Mice received 3 days of training in CFC; on each day, mice were individually placed in the training context (Context A; Figure 6a_1_) for 180 s before receiving a single unsignaled foot shock. As predicted, both Ca_V_1.3^Tg+^ and Ca_V_1.3^Tg−^ mice exhibited an increase in freezing across the 3 days of training, and there were no differences between the two groups (Figure 6a_2_). Twenty‐four hours after training (on Day 4), mice were returned to the training context (Context A) and freezing was measured for 3 min; both Ca_V_1.3^Tg+^ and Ca_V_1.3^Tg−^ mice exhibited robust freezing, indicating successful encoding and retrieval of a fear memory associated with Context A (Figure 6a_3_). On the following day (Day 5), mice were exposed to a novel context (Context C) and freezing levels were recorded over a three‐minute period. When tested in this novel (and what should be considered “safe”) context, Ca_V_1.3^Tg+^ mice froze significantly more than their Ca_V_1.3^Tg−^ littermates, demonstrating an increase in fear generalization.

It has previously been reported that training context re‐exposure impacts subsequent fear generalization to alternate contexts (Huckleberry et al., 2016), which may reflect extinction of the fear associated with the training context. Thus, to rule out this potential confound, we performed a second experiment (Figure 6b_1_) in which a new cohort of mice was trained with the same CFC protocol as above, except that they received three‐foot shocks each day (30 sec intershock interval) for 3 days. As before, this produced an increase in freezing across days of training in both of Ca_V_1.3^Tg‐^ and Ca_V_1.3^Tg+^ mice, with no difference between groups (Figure 6b_2_). Twenty‐four hours after training (on Day 4), instead of being returned to the training context (Context A), mice were put in a new context (Context B) that was similar to, but not the same as, the training context (only the flooring was changed, from a metal grid to a cushioned mat). Freezing was measured for 3 min, during which both Ca_V_1.3^Tg+^ and Ca_V_1.3^Tg−^ mice exhibited robust freezing, indicating that enough contextual features of the training context remained for the mice to demonstrate successful encoding and retrieval of a fear memory (Figure 6b_3_). The next day (Day 5), when exposed to a completely novel context (Context C), Ca_V_1.3^Tg+^ mice still exhibited significantly more freezing compared to their Ca_V_1.3^Tg−^ littermates (Figure 6b_3_). These results corroborate our initial finding demonstrating that Ca_V_1.3^Tg+^ mice exhibit aberrant generalization of fear memories, which was not confounded by the re‐exposure to the training context. This increased fear generalization in young Ca_V_1.3^Tg+^ mice is similar to that observed in aged mice (Figure S2). Both young and aged mice exhibit comparable and significant levels of freezing when tested in the training context (Context A) or a similar context (Context B), but aged mice show higher levels of freezing in a completely novel context (Context C) (Figure S2). This suggests that while young mice are able to discriminate a novel (and safe) context from one associated with an aversive outcome, aged mice show impaired context discrimination and generalize fear to disparate environments.

## 4 DISCUSSION

Calcium signaling is a critical component of many aspects of neuronal function, such as regulating transmitter release (Silva et al., 2021), modulating synaptic plasticity (Giese, 2021), influencing neuronal excitability (Storm, 1987; Thompson et al., 1990), and activating transcription factors, which initiate a variety of downstream pathways (Foster, 2019). Because of its prominent role, the concentration of interneuronal calcium is tightly regulated through the interaction of multiple mechanisms that control calcium entry (via activation of voltage‐gated calcium channels and release from intracellular stores), calcium extrusion (via ATP‐dependent pumps) and calcium buffering (via proteins like calcineurin) (Peterson, 1992). A leading theory regarding the mechanism of brain aging (the “calcium dysregulation hypothesis”) posits that a change in the regulation of interneuronal calcium concentration initiates alterations in neuronal function that ultimately result in cognitive deficits (notably, impairments in learning and memory) (Khachaturian, 1984, 1989).

Experimental evidence has accumulated that supports this hypothesis by demonstrating an age‐dependent increase in interneuronal calcium concentration that appears to be largely mediated by an increase in the number of Ca_V_1.3 L‐VGCCs. For example, electrophysiological experiments have correlated L‐VGCC density and Ca_V_1.3 mRNA levels with increased calcium currents in individual CA1 neurons from aged animals (Chen et al., 2000; Thibault & Landfield, 1996). In addition, molecular biology experiments have shown that both Ca_V_1.3 mRNA and protein levels are increased in CA1 and were inversely correlated with performance in a behavioral task that assesses hippocampus‐dependent learning and memory in the MWM (Chen et al., 2000; Veng & Browning, 2002; Veng et al., 2003). While these data are tantalizing, they are correlational in nature and do not demonstrate the necessity or sufficiency of increased Ca_V_1.3 to age‐related cognitive deficits, particularly in the absence of concomitant changes in other physiological processes that occur with age. Thus, to address this question, we generated a novel transgenic mouse line in which Ca_V_1.3 is overexpressed in forebrain excitatory neurons, (Ca_V_1.3^Tg+^) including those in the CA1 region of the hippocampus (Krueger et al., 2017). Here, we show that this increase in Ca_V_1.3 *in young animals* recapitulates key aspects of age‐related changes in neuronal and cognitive function. Specifically, we observed an increase in the size of the mAHP and sAHP, and a decrease in performance in hippocampus‐dependent learning and memory tasks, elucidating the precise contribution of Ca_V_1.3 to brain aging.

We demonstrate that overexpression of Ca_V_1.3 increases the amplitude of the postburst AHP (particularly the calcium‐dependent medium and slow components), similar to that which is observed in aged mice (Murphy et al., 2004, 2006). Strikingly, however, we find that overexpression of Ca_V_1.3 does not change the current/frequency (I/F) function in CA1 pyramidal neurons, which is in contrast to previous work demonstrating that an increase in the postburst AHP in aged rodents and rabbits is accompanied by a decrease in excitability (fewer action potentials elicited at a given current injection level; for example see [Moyer Jr. et al., 1992; Tombaugh et al., 2005]). While it has been canonically accepted that changes in the AHP alter repetitive action potential firing, there are several studies that have shown that these two properties are not always correlated and can be dissociated from one another. For example, we have reported that genetic ablation of Ca_V_1.3 (Ca_V_1.3 KO) reduces the sAHP but does not increase repetitive firing in CA1 pyramidal neurons (Gamelli et al., 2011). Likewise, corticosterone treatment, which has been shown to increase Ca_V_1.3 mRNA expression in CA1, increases the amplitude of the sAHP but does not significantly change the I/F relationship (Liebmann et al., 2008; Pillai et al., 2014). Our current data are consistent with these observations and further strengthen the hypothesis that alterations in sAHP amplitude can occur without impacting action potential firing rates. Additionally, our results suggest that the change in the I/F relationship exhibited in CA1 neurons from aged animals (specifically, decreased action potential firing) is mediated by age‐related changes in separate, although potentially synergistic, mechanisms.

We also show that overexpression of Ca_V_1.3 in young mice recapitulates deficits in learning and memory paradigms that are observed in aged wild‐type (C57BL/6) mice. In the MWM, aged wild‐type mice perform significantly worse than young wild‐type mice, as evidenced by a significantly longer latency to learn the location of the hidden platform during training, and a nonselective search strategy during probe trials, spending ~25% of the time searching in each quadrant (Murphy et al., 2006). Importantly, if aged mice are given additional training trials, they can eventually form a memory for the platform location and exhibit a selective search strategy (that is, spending significantly >25% of the time in the TQ), similar to the performance of young mice (Murphy et al., 2004). Interestingly, young Ca_V_1.3^Tg+^ mice also show initial deficits in performance (compared to young Ca_V_1.3^Tg−^ that already exhibit a selective search strategy by the first probe trial) that can be overcome with additional training (a selective search strategy in the second probe trial, similar to the performance of Ca_V_1.3^Tg−^). Likewise, young Ca_V_1.3^Tg+^ mice exhibit deficits in the NOR task (evidenced by lack of preference for the novel object, which indicates an absence of memory for the previous exposure to the familiar object), similar to deficits observed in aged mice (Ano et al., 2021; Gosrani et al., 2020; Hendrickx et al., 2022), when a moderate delay (20 min) is imposed. Interestingly, the young Ca_V_1.3^Tg+^ mice exhibit successful memory formation and retrieval (evidenced as a preference for the novel object) when the task is made easier by using a shorter delay (2 min); conversely, aged mice lacking Ca_V_1.3 (Ca_V_1.3 KO) can successfully perform the NOR task with the 20 min delay. Finally, aged mice are prone to overgeneralize fear learning, responding with inappropriate fear‐related behavior (i.e., freezing) in novel contexts that should be perceived as nonaversive (McAvoy et al., 2016; Shoji & Miyakawa, 2019). Reminiscent of this observation, the young Ca_V_1.3^Tg+^ mice also exhibit normal contextual fear conditioning, but do not show significantly reduced freezing in a novel context, suggesting that they also overgeneralize their fear learning.

It is important to note that while the learning and memory deficits exhibited by the Ca_V_1.3^Tg+^ mice parallel those observed in aged mice, overexpression of Ca_V_1.3 does not result in a complete phenocopy. In general, the deficits in aged mice tend to be more severe than those we observe here. For example, compared to Ca_V_1.3^Tg+^ mice, aged mice require significantly more training trials to form a memory for the platform location in MWM (Murphy et al., 2004) and exhibit even worse performance in the NOR task (often showing a negative [instead of neutral] preference for novelty) (Hendrickx et al., 2022). This is perhaps unsurprising given that overexpression of Ca_V_1.3 also only impacts one putative mechanism underlying brain aging (that is, the increased magnitude of the mAHP and sAHP) but does not recapitulate other changes in neuronal function that occur with age (such as the age‐related decrease in action potential firing and alterations in excitatory neurotransmission). In addition, the increased expression of Ca_V_1.3 in these mice was restricted to glutamatergic neurons in the forebrain and hippocampus. Thus, it is likely that increased expression of Ca_V_1.3 in the aged brain is a crucial factor that acts in concert with age‐related changes in other processes to produce the full complement of structural, functional, and behavioral outcomes that are characteristic of aged animals.

Beyond their contribution to cognitive aging and neurodegeneration, L‐VGCCs have long been viewed as a therapeutic target in a wide variety of neurological and psychiatric disease states (Lanzetti & Di Biase, 2022). Currently, there are a number of FDA approved 1,4‐dihydropyridine (DHP) based L‐VGCC inhibitors that are widely used in clinical practice to treat hypertension (Oparil et al., 2018). Unfortunately, these compounds are not subtype specific and have a higher affinity for Ca_V_1.2 (Sinnegger‐Brauns et al., 2009). However, because of its pathological role in Parkinson's disease (Surmeier et al., 2017) there is an ongoing effort to develop Ca_V_1.3 specific antagonists. For example, Kang and colleagues completed a small molecule screen and subsequent hit modification to produce 1‐(3‐chlorophenethyl)‐3‐cyclopentylpyrimidine‐2,4,6‐trione (cp‐PYT), which was found to be highly selective for inhibition of Ca_V_1.3 with a low affinity for Ca_V_1.2 (Kang et al., 2012). Recently, the same group determined the precise binding site of cp‐PYT (Cooper et al., 2020), which will facilitate additional rational design. Future experiments might include pharmacological experiments using optimized cp‐PTY compounds to reverse the enhanced AHP and cognitive deficits observed in the Ca_V_1.3^Tg+^ mice.

## AUTHOR CONTRIBUTIONS

SJM designed experiments, collected and analyzed data, wrote and edited the manuscript; VAC collected and analyzed data, and edited the manuscript; SJT collected and analyzed data; GGM designed experiments, and wrote and edited the manuscript.

## FUNDING INFORMATION

This work was supported by NIH/NIA R01AG074552, U01AG057562, R01AG058171, R01AG052934 and The Protein Folding Disease Initiative, Michigan Medicine, The University of Michigan.

## CONFLICT OF INTEREST

The authors declare that they have no competing interests.

## Supporting information


Figure S1

Figure S2

Appendix S1

## Data Availability

The data that support the findings of this study are available from the corresponding author upon reasonable request.
