# Supplementary figures and images for: Age‐related deficits in neuronal physiology and cognitive function are recapitulated in young mice overexpressing the L‐type calcium channel, CaV1.3
Source: Aging Cell. 2023 Jan 26;22(3):e13781. doi: 10.1111/acel.13781 (PMC10014069; doi:10.1111/acel.13781)

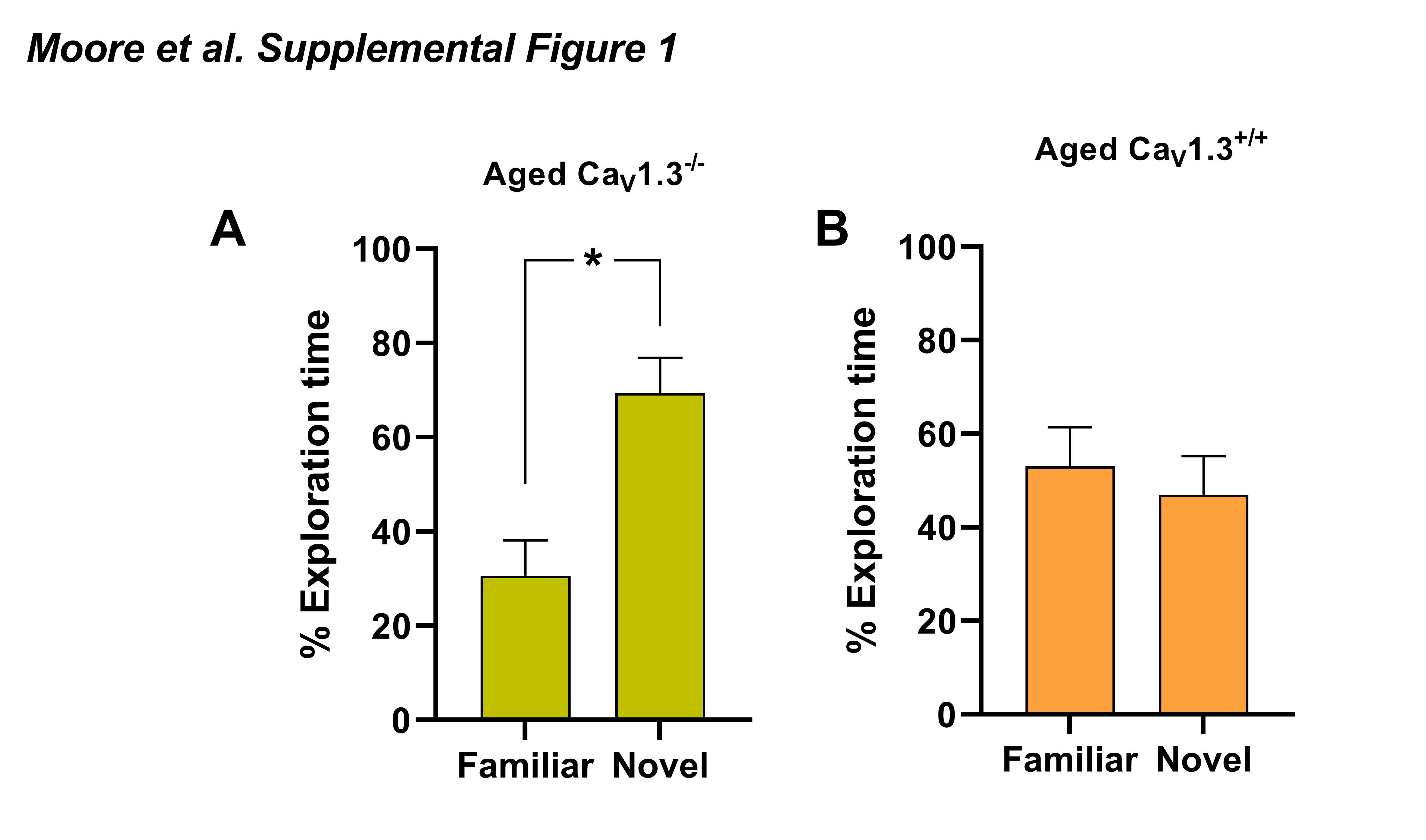

Supplement: Supplementary file 1 — Figure S1 [file ACEL-22-e13781-s003.tif]

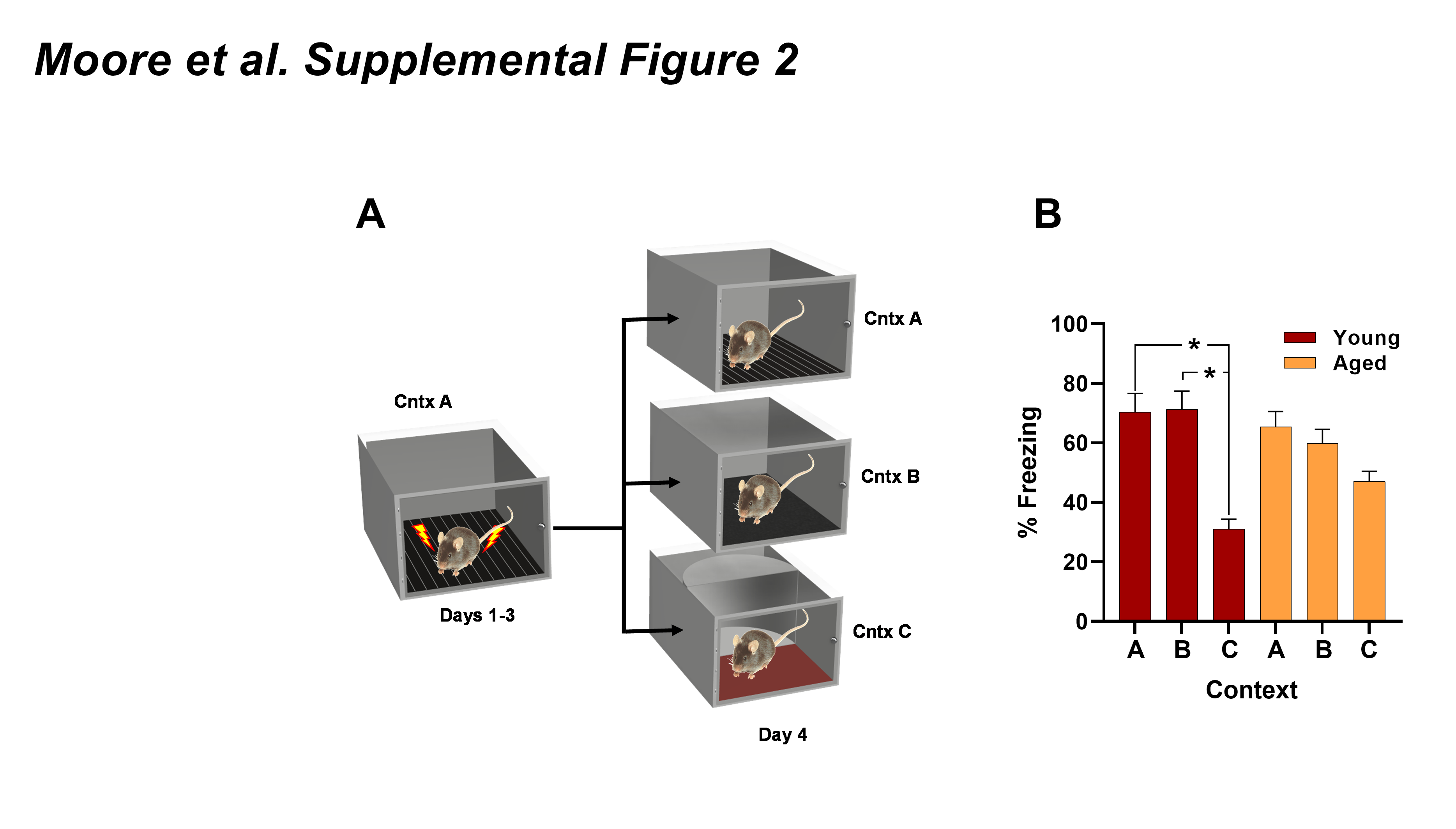

Supplement: Supplementary file 2 — Figure S2 [file ACEL-22-e13781-s001.tif]
