# Supplementary material for: Age‐related deficits in neuronal physiology and cognitive function are recapitulated in young mice overexpressing the L‐type calcium channel, CaV1.3
Source: Aging Cell. 2023 Jan 26;22(3):e13781. doi: 10.1111/acel.13781 (PMC10014069; doi:10.1111/acel.13781)
Supplement: Supplementary file 3 — Appendix S1 [file ACEL-22-e13781-s002.docx]

Supplementary Figure Captions:

FIGURE S1

Deletion of Ca_V_1.3 improves novel object recognition (NOR) in aged (20mo) mice. Aged wild-type (Ca_V_1.3^+/+^, n = 6) and Ca_V_1.3 knockout (Ca_V_1.3^−^^/−^, n = 7) mice were tested after a 20 min delay as in Figure 5. Similar to young Ca_V_1.3^Tg+^ mice, aged wild-type mice spent equal amounts time exploring the familiar and novel objects (p > 0.05, paired t-test), demonstrating that they have an impairment in short-term memory. In contrast, aged Ca_V_1.3^−^^/−^ spent significantly more time exploring the novel object (* indicates p < 0.01, paired t-test), showing that removal of CaV1.3 in aged mice ameliorates the deficit in short-term memory. Data are presented as mean ± sem.

FIGURE S2

Aged mice exhibit increased fear generalization and impaired context generalization. Contextual fear conditioning (CFC) was carried out in young (4 mo; n = 21) and aged (19–20 mo; n = 23) wild-type mice. Mice were placed in the training context (Context A) for 180 s, after which three unsignaled foot shocks (2 s, 0.75 mA, with a 60 s intershock interval) were delivered through the grid floor; mice were remained in the conditioning chambers for an additional 60 s before being returned to their home cage. Twenty-four hours later, mice were divided into separate groups to test fear generalization and context discrimination: one group was returned to the context in which they were trained (Context A), a second group was tested in a context similar to that in which they were trained (Context B, in which the floor was modified from a metal grid to a cushioned mat), and the third group was placed in a completely novel context (Context C, which had a different shape, flooring, odor and lighting). Each group received a three-minute context test during which the amount of time spent freezing was recorded. In young mice, freezing levels were not different in Context A and B, but were significantly lower in Context C (p < 0.001 main effect of context, 1-way ANOVA; * indicate p < 0.05, planned post-hoc comparisons), suggesting these mice could discriminate a very different (safe) context. Conversely, aged mice showed high levels of freeing in all contexts (p > 0.05 main effect of context, 1-way ANOVA), demonstrating that aged mice overgeneralize their learned fear associations and do not effectively discriminate between disparate contexts. Data are presented as mean ± sem.
